# Blood gene expression predicts intensive care unit admission in hospitalised patients with COVID-19

**DOI:** 10.3389/fimmu.2022.988685

**Published:** 2022-09-20

**Authors:** Rebekah Penrice-Randal, Xiaofeng Dong, Andrew George Shapanis, Aaron Gardner, Nicholas Harding, Jelmer Legebeke, Jenny Lord, Andres F. Vallejo, Stephen Poole, Nathan J. Brendish, Catherine Hartley, Anthony P. Williams, Gabrielle Wheway, Marta E. Polak, Fabio Strazzeri, James P. R. Schofield, Paul J. Skipp, Julian A. Hiscox, Tristan W. Clark, Diana Baralle

**Affiliations:** ^1^ Institute of Infection, Veterinary and Ecological Sciences, University of Liverpool, Liverpool, United Kingdom; ^2^ TopMD Precision Medicine Ltd, Southampton, United Kingdom; ^3^ School of Human Development and Health, Faculty of Medicine, University of Southampton, Southampton, United Kingdom; ^4^ National Institute for Health Research (NIHR) Southampton Biomedical Research Centre, University Hospital Southampton National Health Service (NHS) Foundation Trust, University of Southampton, Southampton, United Kingdom; ^5^ School of Clinical and Experimental Sciences, Faculty of Medicine, University of Southampton, Southampton, United Kingdom; ^6^ Cancer Sciences Division, Faculty of Medicine, University Hospital Southampton, Southampton, United Kingdom; ^7^ Institute for Life Sciences, University of Southampton, Southampton, United Kingdom; ^8^ Centre for Proteomic Research, School of Biological Sciences, University of Southampton, Southampton, United Kingdom; ^9^ NIHR Health Protection Research Unit in Emerging and Zoonotic Infections, Liverpool, United Kingdom; ^10^ ASTAR Infectious Diseases Laboratories (ASTAR ID Labs), Agency for Science, Technology and Research (ASTAR) Singapore, Singapore, Singapore

**Keywords:** COVID-19, Critical Care, biomarkers, prognosis, topology, transcriptome, RNA-seq - RNA sequencing

## Abstract

**Background:**

The COVID-19 pandemic has created pressure on healthcare systems worldwide. Tools that can stratify individuals according to prognosis could allow for more efficient allocation of healthcare resources and thus improved patient outcomes. It is currently unclear if blood gene expression signatures derived from patients at the point of admission to hospital could provide useful prognostic information.

**Methods:**

Gene expression of whole blood obtained at the point of admission from a cohort of 78 patients hospitalised with COVID-19 during the first wave was measured by high resolution RNA sequencing. Gene signatures predictive of admission to Intensive Care Unit were identified and tested using machine learning and topological data analysis, TopMD.

**Results:**

The best gene expression signature predictive of ICU admission was defined using topological data analysis with an accuracy: 0.72 and ROC AUC: 0.76. The gene signature was primarily based on differentially activated pathways controlling epidermal growth factor receptor (EGFR) presentation, Peroxisome proliferator-activated receptor alpha (PPAR-α) signalling and Transforming growth factor beta (TGF-β) signalling.

**Conclusions:**

Gene expression signatures from blood taken at the point of admission to hospital predicted ICU admission of treatment naïve patients with COVID-19.

## Introduction

Severe acute respiratory syndrome coronavirus 2 (SARS-CoV-2) is a *betacoronavirus* responsible for coronavirus disease-19 (COVID-19) resulting in a global pandemic with over 6.3 million deaths by June 2022. SARS-CoV-2 causes a spectrum of symptoms in humans, from asymptomatic to severe disease, where the latter requires continuous and intensive care and is associated with extensive pulmonary immunopathology (1, 2). The nature of severe coronavirus disease has caused a strain on healthcare systems across the world (3). Biomarkers predictive of outcome in patients with Ebola virus disease have been identified (4), highlighting that prognostic biomarkers could be useful in outbreak and clinical settings. There is an urgent need for tools which can stratify patients according to prognosis to better manage healthcare resources and improve patient outcomes, particularly in resource poor or limited settings.

There have been many attempts to define prognostic biomarkers in COVID-19 (5–10). However, these have focused on predicting mortality, which is primarily associated with older age groups. The ability to predict, at admission to hospital, the trajectory of a patient towards intensive care unit (ICU) admission will allow for more efficient triaging and improve outcomes through early targeted interventions. The decision to admit individuals to ICU is a result of applying standard clinical and physiological metrics with clinical oversight and scoring tools such as NEWS2 (11). Leveraging host response data from an accessible sample (e.g., peripheral blood) to predict and inform ICU admission is therefore an exciting continuation of previous work to define the host response in patients with COVID-19 (12).

Several studies in different disease contexts, including COVID-19, have been conducted to predict in-hospital mortality and ICU admission (11, 13–17). For sepsis, NEWS, was assessed for the prediction of in-hospital death with an AUROC of 0.65 (0.61 to 0.68) and ICU admission with an AUROC of 0.64 (0.57 to 0.71), however, the authors highlight that no scoring system has both high sensitivity and specificity for predicting adverse outcomes in sepsis at admission (13). A retrospective analysis of data available at the time of admission, including heart rate, supplementary oxygen, abnormal sodium, and amount of time spent in the emergency department, was used to build a logistic regression model to predict early ICU admission which produced a AUROC of 0.70 (0.67-0.72), and was able to identify 10% of early ICU transfers (14). Some attempts in predicting COVID-19 ICU admission have not performed well (16). However, a model based on age, sex and comorbidities did predict ICU mortality and ICU admission in COVID-19 patients, generating a c-statistic of 0.876 (0.864-0.886) (11). Others have found that CURB-65 scores perform well in predicting in-hospital mortality with an AUC of 0.781, and the qCSI score performed well in predicting ICU admission with an AUC of 0.761 (15). Models with AUC values between 0.86 – 0.88 have been developed for predicting hospitalisation, ICU care and mechanical ventilation (18). Age and BMI were important predictors for hospitalisation, whereas for ICU admission male sex, opacities in chest scans and age were important variables (17). Routine laboratory values predictive of ICU admission and mechanical ventilation included elevated serum lactate dehydrogenase (LDH), C-reactive protein (CRP), anion gap and glucose, in addition to decreased serum calcium, sodium and albumin (17).

Using gene expression signatures to predict clinical outcome or care trajectories, from a sample such as blood, have been infrequently reported in the literature. Previously, an 11-gene host response score was found to perform similarly to SAPS3 and APACHE II as a stand-alone test, from whole blood collected within 30 days of admission when predicting 60-day mortality (AUC: 0.68), in-hospital mortality (AUC: 0.75), shock patients (AUC: 77) and primary MODS or ARDS (AUC: 0.98) (19). In sepsis, 20 and 10 gene panels have been trialed with AUCs between 0.723 to 0.956 being achieved depending on the cohort and the number of genes included in the panel (20).

Genomic analyses such as RNAseq are routinely used to inform clinical decisions (21–24). Turnaround times from sampling to actionable data are continually improving, making their potential use as point-of-care tools more feasible. In addition, the cost of sequencing continues to decrease and many sequencing platforms are becoming more accessible. In this study, using blood gene expression profiles from 78 SARS-CoV-2 infected patients, machine learning and an emerging topological data analysis approach (25, 26) was used to identify and validate gene signatures that were predictive of ICU admission of patients with COVID-19 disease. This predictive model, demonstrates potential as a valuable tool for personalised treatment and assist in the clinical decision making for hospitalised COVID-19 patients, and provide a point of comparison for evaluating the effects of medical countermeasures.

## Materials and methods

### Patient cohort and study design

In this study, a cohort of 78 patients presenting hospitalised with COVID-19 were analysed. Samples were collected as part of the CoV-19POC study (ISRCTN trial registry: ISRCTN14966673) as previously described (12). In brief, blood samples were collected in PAXgene tubes within 24 hours of admission to hospital between March and April 2020. All patients were sampled and RNAseq data generated. Detailed patient characteristics and demographics collected at time of admission from medical records, are included in **Table 1**, generated by gtsummary (27).

**Table 1:** Patient characteristics and demographics grouped by ICU admission status.

Characteristic	N	Overall, N = 78^1^	Not admitted to ICU, N = 51^1^	Admitted to ICU, N = 27^1^	p-value^2^
**Dataset split**	78				0.43
** Test**		48 (62%)	33 (65%)	15 (56%)	
** Training**		30 (38%)	18 (35%)	12 (44%)	
**Symptom duration days**	78	7 (5, 10)	7 (4, 10)	8 (6, 10)	0.43
**LOS (hours)**	75	198 (100, 332)	147 (59, 256)	296 (214, 546)	<0.001
**Age**	78	61 (46, 74)	70 (49, 80)	56 (45, 61)	0.006
**Sex**	78				0.61
** F**		26 (33%)	18 (35%)	8 (30%)	
** M**		52 (67%)	33 (65%)	19 (70%)	
**Smoking status**	78				0.91
** Ex-smoker**		31 (40%)	21 (41%)	10 (37%)	
** Never**		36 (46%)	22 (43%)	14 (52%)	
** Unknown**		7 (9.0%)	5 (9.8%)	2 (7.4%)	
** Yes**		4 (5.1%)	3 (5.9%)	1 (3.7%)	
**Ethnicity**	78				0.037
** White - British**		47 (60%)	33 (65%)	14 (52%)	
** White – Any other white background**		6 (7.7%)	6 (12%)	0 (0%)	
** Asian or Asian British - Indian**		3 (3.8%)	2 (3.9%)	1 (3.7%)	
** Asian or Asian British – Any other Asian background**		13 (17%)	5 (9.8%)	8 (30%)	
** Black or Black British - Caribbean**		2 (2.6%)	2 (3.9%)	0 (0%)	
** Black or Black British - African**		6 (7.7%)	2 (3.9%)	4 (15%)	
** Unknown**		1 (1.3%)	1 (2.0%)	0 (0%)	
**Clinical Metrics**					
**Hypertension**	78				0.72
** No**		45 (58%)	29 (57%)	16 (59%)	
** Unknown**		4 (5.1%)	2 (3.9%)	2 (7.4%)	
** Yes**		29 (37%)	20 (39%)	9 (33%)	
**CV disease**	78				0.17
** No**		59 (76%)	37 (73%)	22 (81%)	
** Unknown**		3 (3.8%)	1 (2.0%)	2 (7.4%)	
** Yes**		16 (21%)	13 (25%)	3 (11%)	
**Resp Disease other**	78				0.47
** No**		54 (69%)	35 (69%)	19 (70%)	
** Unknown**		3 (3.8%)	1 (2.0%)	2 (7.4%)	
** Yes**		21 (27%)	15 (29%)	6 (22%)	
**Asthma**	78				0.51
** No**		62 (79%)	41 (80%)	21 (78%)	
** Unknown**		3 (3.8%)	1 (2.0%)	2 (7.4%)	
** Yes**		13 (17%)	9 (18%)	4 (15%)	
**COPD**	78				0.49
** No**		68 (87%)	45 (88%)	23 (85%)	
** Unknown**		3 (3.8%)	1 (2.0%)	2 (7.4%)	
** Yes**		7 (9.0%)	5 (9.8%)	2 (7.4%)	
**CKD**	78				0.53
** No**		69 (88%)	46 (90%)	23 (85%)	
** Unknown**		3 (3.8%)	1 (2.0%)	2 (7.4%)	
** Yes**		6 (7.7%)	4 (7.8%)	2 (7.4%)	
**CLD**	78				0.55
** No**		72 (92%)	48 (94%)	24 (89%)	
** Unknown**		3 (3.8%)	1 (2.0%)	2 (7.4%)	
** Yes**		3 (3.8%)	2 (3.9%)	1 (3.7%)	
**Diabetes**	78				0.50
** No**		56 (72%)	38 (75%)	18 (67%)	
** Unknown**		3 (3.8%)	1 (2.0%)	2 (7.4%)	
** Yes**		19 (24%)	12 (24%)	7 (26%)	
**Active malignancy**	78				0.11
** No**		69 (88%)	44 (86%)	25 (93%)	
** Unknown**		3 (3.8%)	1 (2.0%)	2 (7.4%)	
** Yes**		6 (7.7%)	6 (12%)	0 (0%)	
**Dementia**	78				0.048
** No**		67 (86%)	42 (82%)	25 (93%)	
** Unknown**		3 (3.8%)	1 (2.0%)	2 (7.4%)	
** Yes**		8 (10%)	8 (16%)	0 (0%)	
**Immunosuppressed^+^**	78				0.16
** No**		70 (90%)	48 (94%)	22 (81%)	
** Unknown**		4 (5.1%)	1 (2.0%)	3 (11%)	
** Yes**		4 (5.1%)	2 (3.9%)	2 (7.4%)	
**Abx during admission**	78	72 (92%)	46 (90%)	26 (96%)	0.66
**Blood chemistry**					
**Haemoglobin**	77	132 (124, 148)	132 (122, 140)	144 (128, 151)	0.20
**White blood cells**	77	7.8 (5.7, 11.5)	6.2 (4.9, 10.7)	9.4 (6.8, 11.5)	0.026
**Platelets**	77	236 (184, 291)	227 (182, 280)	255 (186, 294)	0.45
**Neutrophils**	77	6.0 (4.0, 9.6)	4.8 (3.6, 8.1)	8.2 (5.2, 9.7)	0.012
**Lymphocytes**	77	1.00 (0.80, 1.20)	1.00 (0.80, 1.18)	1.00 (0.75, 1.30)	0.72
**Sodium**	77	135 (133, 138)	136 (133, 138)	135 (132, 136)	0.091
**Potassium**	71	4.00 (3.65, 4.40)	3.90 (3.65, 4.35)	4.00 (3.75, 4.43)	0.44
**Urea**	77	6.3 (4.7, 9.9)	6.4 (4.3, 10.1)	6.3 (4.8, 8.9)	0.95
**Creatine**	77	86 (67, 110)	84 (67, 109)	88 (66, 108)	0.79
**Albumin**	73	34 (30, 35)	34 (31, 36)	31 (28, 34)	0.009
**Bilirubin**	73	11 (8, 13)	10 (8, 12)	11 (10, 14)	0.063
**Alanine Aminotransferase**	70	34 (24, 70)	28 (21, 56)	37 (32, 77)	0.050
**Alkaline Phosphatase**	73	89 (61, 115)	80 (56, 110)	96 (62, 124)	0.12
**Total Protein**	72	70 (66, 73)	70 (67, 73)	71 (66, 74)	0.85
**LDH**	52	766 (561, 1,133)	650 (506, 776)	1,130 (850, 1,382)	<0.001
**Ferritin**	61	678 (354, 1,693)	521 (203, 750)	1,427 (908, 2,073)	<0.001
**D-dimer**	45	469 (320, 942)	456 (298, 1,426)	535 (385, 812)	0.53
**Trop**	60	12 (5, 46)	11 (4, 28)	12 (8, 63)	0.25
**CRP**	77	120 (52, 164)	80 (22, 131)	168 (128, 254)	<0.001
**Cytokines/Chemokines**					
**IL-6 (pg/mL)**	78	52 (32, 106)	43 (30, 85)	82 (43, 132)	0.030
**TNFα (pg/mL)**	78	20 (17, 25)	20 (16, 25)	22 (19, 25)	0.27
**IL-8 (pg/mL)**	78	35 (26, 59)	34 (25, 53)	49 (34, 64)	0.10
**IL-1β9 (pg/mL)**	78	0.39 (0.26, 0.56)	0.35 (0.24, 0.48)	0.47 (0.31, 0.66)	0.056
**GM-CSF (pg/mL)**	78	1.30 (0.80, 1.86)	1.20 (0.77, 2.28)	1.49 (1.01, 1.79)	0.43
**IFNg (pg/mL)**	78	12 (4, 27)	9 (2, 27)	16 (8, 35)	0.072
**IL-10 (pg/mL)**	78	16 (10, 28)	15 (8, 27)	17 (13, 29)	0.22
**IL-33 (pg/mL)**	78	0.35 (0.17, 0.61)	0.28 (0.15, 0.39)	0.46 (0.35, 0.91)	0.009
**Physiological Metrics**					
**Heart Rate**	78	98 (85, 109)	92 (82, 107)	102 (94, 110)	0.064
**Systolic Blood Pressure**	78	132 (122, 143)	135 (122, 145)	128 (124, 134)	0.14
**Respiration rate**	78	26 (20, 32)	24 (20, 28)	28 (23, 34)	0.054
**Oxygen Saturation**	78	95 (92, 96)	96 (93, 97)	95 (90, 96)	0.047
**Temperature (°C)**	76	37.20 (36.60, 38.20)	37.20 (36.65, 38.10)	37.20 (36.60, 38.40)	0.61
**O2**	78	37 (47%)	17 (33%)	20 (74%)	<0.001
**NEWS2**	76	6 (4, 7)	5 (2, 6)	6 (5, 8)	0.012
**CXR**	78	77 (99%)	50 (98%)	27 (100%)	>0.99
**Consolidation or infiltrates**	77	66 (86%)	39 (78%)	27 (100%)	0.007
**CT**	78	8 (10%)	5 (9.8%)	3 (11%)	>0.99
**ICU specific metrics**					
**Duration O2**	78	19 (5, 112)	9 (1, 17)	161 (110, 397)	<0.001
**NIV duration**	78	0 (0, 13)	0 (0, 0)	25 (6, 49)	<0.001
**IV duration**	78	0 (0, 0)	0 (0, 0)	114 (0, 277)	<0.001
**Optiflow duration**	78	7 (9.0%)	0 (0%)	7 (26%)	<0.001
**ECMO**	75	1 (1.3%)	0 (0%)	1 (4.2%)	0.32
**Died within 30 days of admission**	77	15 (19%)	12 (24%)	3 (12%)	0.21

^1^n (%); Median (IQR) ^2^ arson’s Chi-squared test; Wilcoxon rank sum test; Fisher’s exact test.

^+^Immunosuppressed definition derived from UKHSA Influenza treatment guidance.

ICU, Intensive Care Unit; CV, Cardiovascular; COPD, Chronic obstructive pulmonary disease; CKD, Chronic kidney disease; CLD, Chronic liver disease; LDH, Lactate dehydrogenase; IL, Interleukin; TNF, Tumour necrosis factor; GMCSF, Granulocyte-macrophage colony-stimulating factor; IFN, Interferon; NEWS2, National early warning score 2; O2, administration of supplementary oxygen; CXR, Chest x-ray; CT, Computational tomography; NIV, Non-invasive ventilation; IV, Invasive ventilation; ECMO, Extracorporeal membrane oxygenation.

### Extraction of RNA from clinical samples and Illumina sequencing

Total RNA was extracted from PAXgene BRT using the PAXgene Blood RNA Kit (PreAnalytix), according to the manufactures protocol at Containment Level 3 in a Tripass Class I hood. Libraries were sequenced using 150 bp paired-end reads on an Illumina^®^ NovaSeq 6000.

### Data processing and machine learning

Raw paired-end fastq files generated by the NovaSeq were trimmed for the presence of adapter sequences using cutadapt (v.1.2.1), with the -O 3 parameter (28). The fastq files were further trimmed using sickle (v.1.200) with a minimum window quality score of 20 and reads shorter than 15bp are removed from analysis (18). Hisat2 v2.1.0 (29) was used to map the trimmed reads on the reference Homo sapiens genome assembly (release-94) downloaded from the Ensembl FTP site. The resultant alignment files were processed by featureCounts v2.0.0 (30) with the default setting to generate raw read counts per gene. Before further analysis, outlier samples in the hierarchical clustering were removed and low-expression genes (at least 1 read per million in smallest groups) were filtered. The decision trees models to classify ICU admission in COVID-19 samples were built according to the random forest classifier based on gene expression or traits of hospital assay by using randomForest() function in R package “randomForest” (31) with “ntree=500, proximity=TRUE, mtry=5”. Variable importance in the random forest models were measured through mean decrease in accuracy and the Gini Index.

### Topological data analysis (TDA)

To determine reliability and accuracy of the TDA method presented here, the cohort was divided randomly in two not-overlapping sets, one for training (48 samples) and another for statistical testing (30 samples). Patient demographics and characteristics are presented in **Table 2** for the test and training datasets. The average gene expression of ICU samples within the training set was also calculated and its topology of the global differential gene expression was measured by Topological Pathway Mapping, TopMD, without filtering. Such topology was then used as a reference with respect to the topology of global differential gene expression of each sample. Highly modulated pathways are large features of the TopMD Maps; gene pathways of high importance. When performing the regression analysis, *via* Logistic Regression with ElasticNet penalty (see formula below), we stress that the TopMD ICU profile used as reference was computed only on the training set.

**Table 2:** Patient characteristics and demographics grouped by test or train status.

Characteristic	N	Overall, N = 78^1^	Test, N = 48^1^	Training, N = 30^1^	p-value^2^
**Symptom duration days**	78	7 (5, 10)	8 (6, 10)	6 (2, 10)	0.076
**Length of stay (hours)**	75	198 (100, 332)	206 (104, 331)	186 (80, 376)	0.98
**Age (years)**	78	61 (46, 74)	56 (41, 72)	66 (58, 76)	0.028
**Sex**	78				0.62
** F**		26 (33%)	15 (31%)	11 (37%)	
** M**		52 (67%)	33 (69%)	19 (63%)	
**Smoking status**	78				0.90
** Ex-smoker**		31 (40%)	19 (40%)	12 (40%)	
** Never**		36 (46%)	21 (44%)	15 (50%)	
** Unknown**		7 (9.0%)	5 (10%)	2 (6.7%)	
** Yes**		4 (5.1%)	3 (6.2%)	1 (3.3%)	
**Ethnicity**	78				0.034
** White - British**		47 (60%)	27 (56%)	20 (67%)	
** White – Any other white background**		6 (7.7%)	5 (10%)	1 (3.3%)	
** Asian or Asian British - Indian**		3 (3.8%)	1 (2.1%)	2 (6.7%)	
** Asian or Asian British – Any other Asian background**		13 (17%)	11 (23%)	2 (6.7%)	
** Black or Black British - Caribbean**		2 (2.6%)	2 (4.2%)	0 (0%)	
** Black or Black British - African**		6 (7.7%)	1 (2.1%)	5 (17%)	
** Unknown**		1 (1.3%)	1 (2.1%)	0 (0%)	
**Clinical Metrics**					
**Hypertension**	78				0.008
** No**		45 (58%)	34 (71%)	11 (37%)	
** Unknown**		4 (5.1%)	2 (4.2%)	2 (6.7%)	
** Yes**		29 (37%)	12 (25%)	17 (57%)	
**CV disease**	78				0.55
** No**		59 (76%)	38 (79%)	21 (70%)	
** Unknown**		3 (3.8%)	1 (2.1%)	2 (6.7%)	
** Yes**		16 (21%)	9 (19%)	7 (23%)	
**Resp Disease other**	78				0.39
** No**		54 (69%)	32 (67%)	22 (73%)	
** Unknown**		3 (3.8%)	1 (2.1%)	2 (6.7%)	
** Yes**		21 (27%)	15 (31%)	6 (20%)	
**Asthma**	78				0.71
** No**		62 (79%)	39 (81%)	23 (77%)	
** Unknown**		3 (3.8%)	1 (2.1%)	2 (6.7%)	
** Yes**		13 (17%)	8 (17%)	5 (17%)	
**COPD**	78				0.22
** No**		68 (87%)	41 (85%)	27 (90%)	
** Unknown**		3 (3.8%)	1 (2.1%)	2 (6.7%)	
** Yes**		7 (9.0%)	6 (12%)	1 (3.3%)	
**CKD**	78				0.018
** No**		69 (88%)	46 (96%)	23 (77%)	
** Unknown**		3 (3.8%)	1 (2.1%)	2 (6.7%)	
** Yes**		6 (7.7%)	1 (2.1%)	5 (17%)	
**CLD**	78				0.80
** No**		72 (92%)	45 (94%)	27 (90%)	
** Unknown**		3 (3.8%)	1 (2.1%)	2 (6.7%)	
** Yes**		3 (3.8%)	2 (4.2%)	1 (3.3%)	
**Diabetes**	78				0.048
** No**		56 (72%)	39 (81%)	17 (57%)	
** Unknown**		3 (3.8%)	1 (2.1%)	2 (6.7%)	
** Yes**		19 (24%)	8 (17%)	11 (37%)	
**Active malignancy**	78				0.48
** No**		69 (88%)	44 (92%)	25 (83%)	
** Unknown**		3 (3.8%)	1 (2.1%)	2 (6.7%)	
** Yes**		6 (7.7%)	3 (6.2%)	3 (10%)	
**Dementia**	78				0.45
** No**		67 (86%)	43 (90%)	24 (80%)	
** Unknown**		3 (3.8%)	1 (2.1%)	2 (6.7%)	
** Yes**		8 (10%)	4 (8.3%)	4 (13%)	
**Immunosuppressed** ^+^	78				0.004
** No**		70 (90%)	47 (98%)	23 (77%)	
** Unknown**		4 (5.1%)	1 (2.1%)	3 (10%)	
** Yes**		4 (5.1%)	0 (0%)	4 (13%)	
**Abx during admission**	78	72 (92%)	44 (92%)	28 (93%)	>0.99
**Blood Chemistry**					
**Haemoglobin**	77	132 (124, 148)	132 (124, 152)	132 (127, 144)	0.39
**White blood cells**	77	7.8 (5.7, 11.5)	7.1 (5.6, 11.2)	9.0 (6.0, 11.7)	0.32
**Platelets**	77	236 (184, 291)	228 (181, 289)	260 (191, 291)	0.45
**Neutrophils**	77	6.0 (4.0, 9.6)	4.9 (3.8, 8.5)	7.4 (4.4, 9.7)	0.14
**Lymphocytes**	77	1.00 (0.80, 1.20)	1.00 (0.80, 1.20)	1.00 (0.70, 1.20)	0.56
**Sodium**	77	135 (133, 138)	136 (134, 138)	134 (133, 136)	0.011
**Potassium**	71	4.00 (3.65, 4.40)	3.95 (3.75, 4.40)	4.00 (3.65, 4.35)	0.94
**Urea**	77	6.3 (4.7, 9.9)	5.8 (4.3, 9.5)	7.2 (5.8, 9.9)	0.20
**Creatine**	77	86 (67, 110)	84 (66, 102)	87 (76, 125)	0.47
**Albumin**	73	34 (30, 35)	34 (31, 36)	31 (28, 34)	0.012
**Bilirubin**	73	11 (8, 13)	11 (8, 14)	10 (8, 13)	0.71
**Alanine Aminotransferase**	70	34 (24, 70)	34 (26, 65)	36 (22, 75)	0.57
**Alkaline Phosphatase**	73	89 (61, 115)	93 (61, 117)	84 (62, 106)	0.54
**Total Protein**	72	70 (66, 73)	70 (67, 75)	69 (66, 72)	0.41
**LDH**	52	766 (561, 1,133)	698 (540, 932)	1,022 (661, 1,380)	0.061
**Ferritin**	61	678 (354, 1,693)	638 (421, 1,291)	970 (339, 1,978)	0.51
**D-dimer**	45	469 (320, 942)	448 (350, 886)	535 (300, 884)	0.89
**Trop**	60	12 (5, 46)	10 (5, 50)	13 (9, 34)	0.33
**CRP**	77	120 (52, 164)	92 (44, 155)	135 (108, 185)	0.046
**Cytokines/Chemokines**					
**IL-6 (pg/ml)**	78	52 (32, 106)	41 (25, 85)	82 (44, 174)	0.002
**TNFα (pg/ml)**	78	20 (17, 25)	20 (15, 24)	21 (18, 28)	0.090
**IL-8(pg/ml)**	78	35 (26, 59)	34 (21, 52)	49 (32, 72)	0.006
**IL-1β9 (pg/ml)**	78	0.39 (0.26, 0.56)	0.36 (0.24, 0.49)	0.46 (0.29, 0.61)	0.20
**GM-CSF (pg/ml)**	78	1.30 (0.80, 1.86)	1.21 (0.76, 1.65)	1.47 (0.82, 2.58)	0.20
**IFNg (pg/ml)**	78	12 (4, 27)	13 (2, 27)	11 (6, 28)	0.73
**IL-10 (pg/ml)**	78	16 (10, 28)	14 (8, 24)	20 (14, 31)	0.052
**IL-33(pg/ml)**	78	0.35 (0.17, 0.61)	0.34 (0.17, 0.51)	0.36 (0.17, 0.69)	0.50
**Physiological Metrics**					
**Heart Rate**	78	98 (85, 109)	102 (88, 110)	90 (85, 100)	0.034
**Systolic Blood Pressure**	78	132 (122, 143)	130 (120, 138)	134 (126, 145)	0.20
**Respiration Rate**	78	26 (20, 32)	24 (20, 32)	26 (22, 33)	0.17
**Oxygen Saturation**	78	95 (92, 96)	96 (93, 97)	94 (91, 96)	0.051
**Temperature (°C)**	76	37.20 (36.60, 38.20)	37.20 (36.60, 38.23)	37.20 (36.68, 38.12)	0.88
**O2**	78	37 (47%)	18 (38%)	19 (63%)	0.026
**NEWS2**	76	6 (4, 7)	6 (3, 7)	6 (5, 6)	0.62
**CXR**	78	77 (99%)	48 (100%)	29 (97%)	0.38
**Consolidation or infiltrates**	77	66 (86%)	40 (83%)	26 (90%)	0.52
**CT**	78	8 (10%)	6 (12%)	2 (6.7%)	0.70
**ICU Specific Metrics**					
Duration O2*	78	19 (5, 112)	16 (4, 88)	24 (14, 179)	0.091
**ICU admission**	78	27 (35%)	15 (31%)	12 (40%)	0.43
NIV duration*	78	0 (0, 13)	0 (0, 16)	0 (0, 9)	0.93
IV duration*	78	0 (0, 0)	0 (0, 0)	0 (0, 151)	0.030
Optiflow duration*	78	7 (9.0%)	3 (6.2%)	4 (13%)	0.42
ECMO*	75	1 (1.3%)	1 (2.1%)	0 (0%)	>0.99
Died within 30 days of admission*	77	15 (19%)	6 (12%)	9 (31%)	0.047

^1^Median (IQR); n (%) ^2^Wilcoxon rank sum test; Pearson’s Chi-squared test; Fisher’s exact test.

* Metric excluded from machine learning analysis.

^+^Immunosuppressed definition derived from UKHSA Influenza treatment guidance.

ICU, Intensive Care Unit; CV, Cardiovascular; COPD, Chronic obstructive pulmonary disease; CKD, Chronic kidney disease; CLD, Chronic liver disease; LDH, Lactate dehydrogenase; IL, Interleukin; TNF, Tumour necrosis factor; GMCSF, Granulocyte-macrophage colony-stimulating factor; IFN, Interferon; NEWS2, National early warning score 2; O2, administration of supplementary oxygen; CXR, Chest x-ray; CT, Computational tomography; NIV, Non-invasive ventilation; IV, Invasive ventilation; ECMO, Extracorporeal membrane oxygenation.

To define a gene signature, TopMD profiles were computed for both each patient blood sample and the ICU average gene expression within the training set, relative to the average of all training set samples. From the training ICU profile, a panel of *m* genes taken from *N* TopMD-pathways of highest importance was selected and subsequently a feature matrix was constructed to perform the linear regression analysis, as follows.

From the training ICU profile, a reference panel is constructed using the most important *N* TopMD-pathways and, per each of them, the *m* most abundant genes. The feature matrix was then constructed associating each sample to a row and each reference gene to a column, that is, the entry (i, j) referred to sample Pi and gene gj. Any matrix entry (i, j) was defined to be 0 whenever the gene gj was not within the TopMD-defined sample panel, that is, gj was not one of the *m* most abundant genes within the *N* most important TopMD-pathways for the Pi TopMD-profile. Otherwise, such entry was the relative gene expression of gj for sample Pi.

For the statistical analysis, the Logistic Regression model, with ElasticNet penalty, was used, defined by the following formula:


$$\underset{w,c}{\min}\frac{1-p}{2}w^{T}w+p{\left||w|\right|}_{1}+C\sum_{i=1}^{n}\log(\exp(-y_{i}(X_{i}^{T}w+c))+1)$$

Where X is the feature matrix, y the binary classification vector and w is the weights vector. Parameters for this model are C, a regularisation parameter (improving numerical stability), and ρ which controls the strength of l1 and l2 regularisation, respectively the first and second member in the formula. The best performing panel of genes was selected, among all the combination of *N* and *m* with value ranging from 1 to 100, given that *m*≤*N*. The best performing model, with respect to predictive error, was obtained using *N*=10 TopMD-pathways and *m*=5 genes. The regression model allows naturally to define the belongingness probability to the positive class, the ICU class in this case. For statistical testing purposes, each patient blood sample in the test set is predicted to be ICU when such probability is higher than 0.5.

### Statistical analysis

Statistical testing was performed including a Shapiro-Wilk test to assess for data normality followed with either an unpaired parametric T-test (Shapiro-Wilk test p-value > 0.05) or an unpaired non-parametric Wilcoxon test (Shapiro-Wilk test p-value< 0.05) for continuous data, or a Chi-square test for categorical data.

## Results

To identify transcripts that were predictive of ICU admission for those with COVID-19 disease, the transcriptome of blood samples from infected patients was analysed.

### Patient characteristics

These samples were collected through the CoV-POC trial in early 2020. Out of the 78 samples included in this study, 48 were included in the training dataset and 30 in the test dataset. The median age of the study population was 61 (IQR: 46-74) 52 were male (67%) and 26 were female (33%). The most common comorbidities were hypertension (37%), chronic respiratory disease (27%) and diabetes mellitus (24%) (**Table 1**). 27 were admitted to ICU of which 15 died within 30 days of admission. In this dataset there was no difference in sex between those admitted to ICU and those not admitted to ICU, p = 0.61. Age was different between those admitted to ICU and those not admitted to ICU, p 0.006, median age of 56 and 70 years respectively. **Table 1** shows that data points from blood chemistry, cytokine/chemokine assessment and physiological metrics are significantly different between the patients admitted to ICU and not admitted to ICU. Including white blood cell count, neutrophil count, albumin, LDH, ferritin, CRP, IL-6, IL-33, Oxygen saturation, administration of oxygen, NEWS and consolidation of infiltrates. Patient characteristics and demographics are also shown for the test and training split (**Table 2**).

### Machine learning

**Figure 1:**
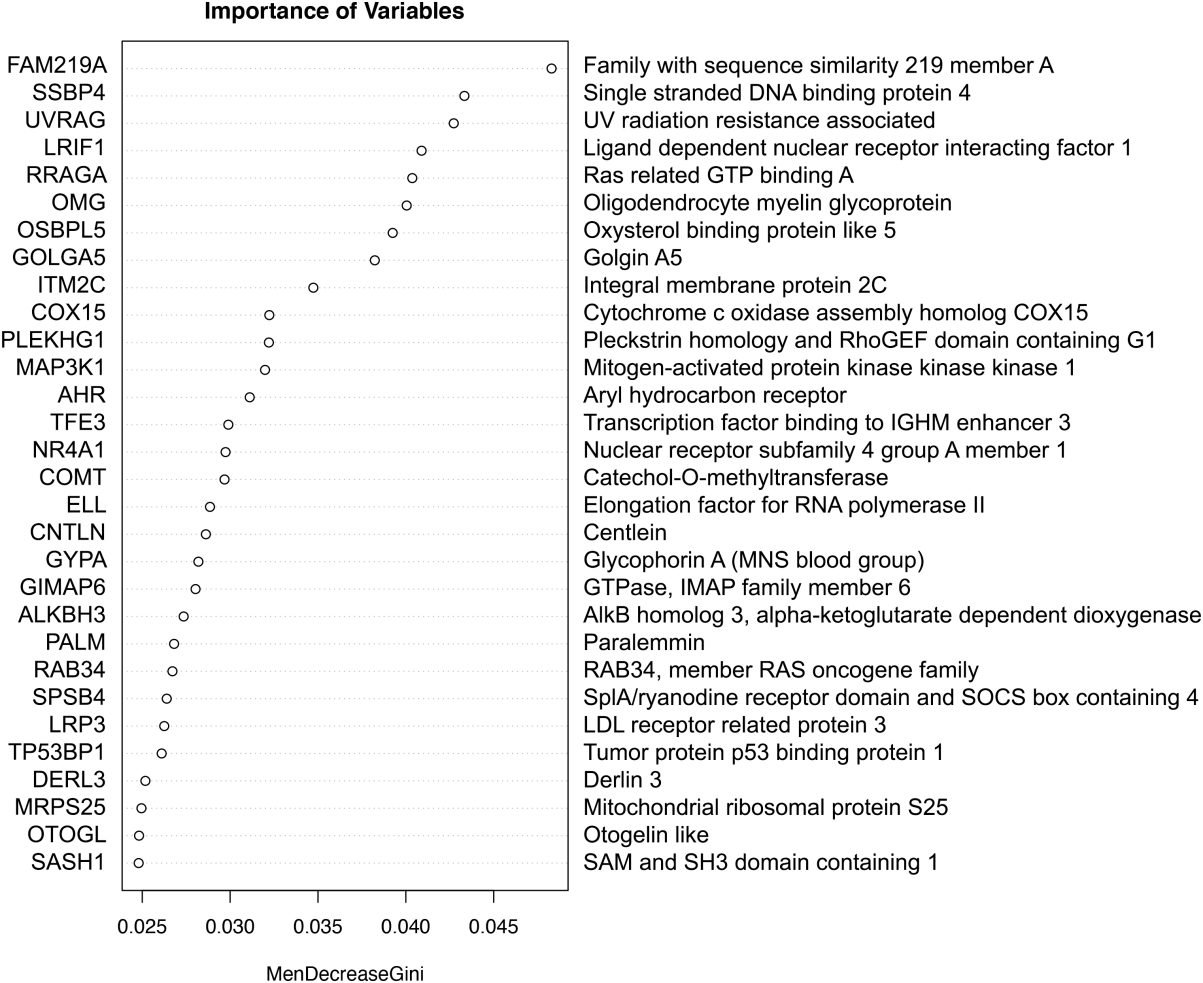
The importance of genes in a classification of ICU admission with Random Forest. The higher the value of importance of the variable (mean decrease gini score), the higher the importance of the gene(s) in the model.

Combinations of the top 30 important genes were identified by Random Forest analysis predictive of ICU admission in the test dataset (**Figure 1**), achieving good accuracy (0.73) and a ROC of 0.68. The higher the value of importance of the variable (mean decrease gini score), the higher the importance of the genes in the model. In this analysis, the gene that was most associated with the decision to admit to ICU was family with sequence similarity 219 member A (FAM219A) gene.

### Topological data analysis

**Figure 2:**
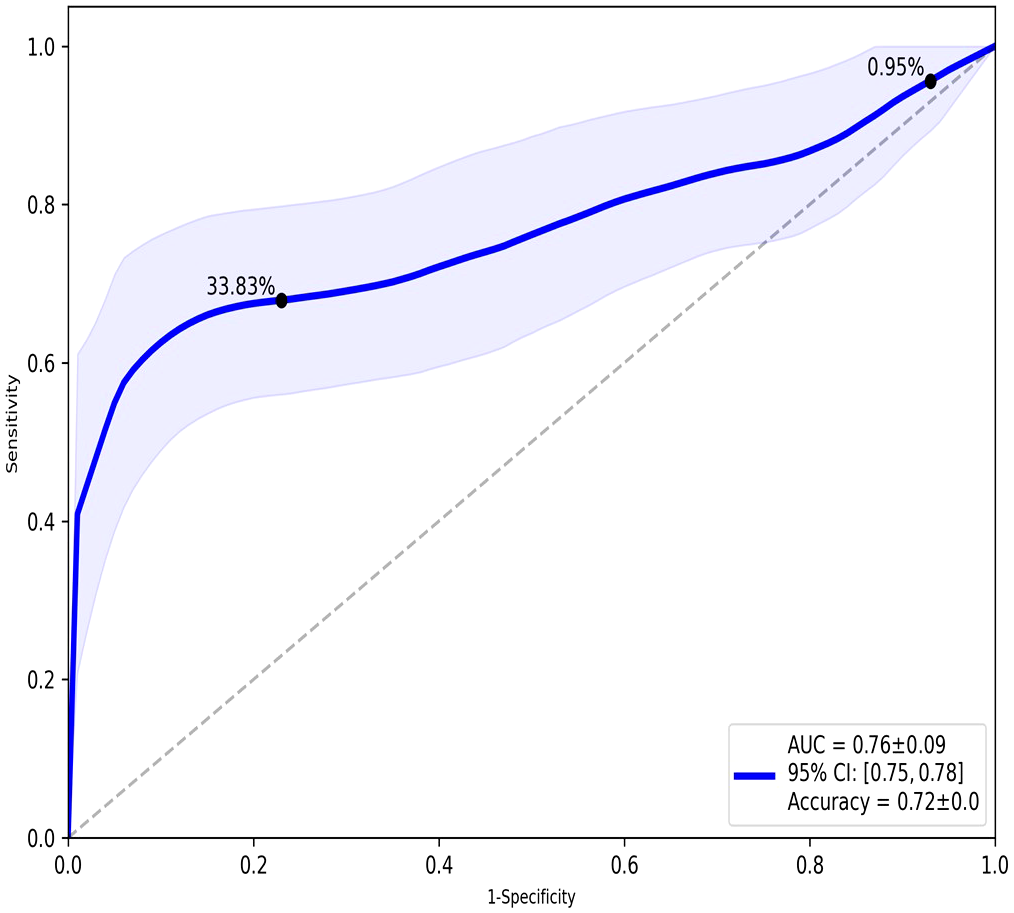
ROC analysis of the overall performance of the TopMD-defined gene signature predictive of ICU admission. ROC curve with split 62/38, using top 10 pathways with top 10 genes for a total of 79 genes overall.

TopMD Pathway Biomarker Analysis defined a model with 79 genes identified from TopMD clusters predictive of ICU admission in the test dataset with accuracy: 0.72 and ROC AUC: 0.76 (**Figure 2**). The genes of this predictive signature were features of the top 10 pathways with top 10 genes for a total of 79 genes overall; differentially activated gene pathways between patients admitted to ICU or not admitted to ICU in the training dataset.

### The top 3 identified pathways predictive of ICU admission are involved in EGFR, PPAR-α and TGFβ signalling pathways

**Figure 3:**
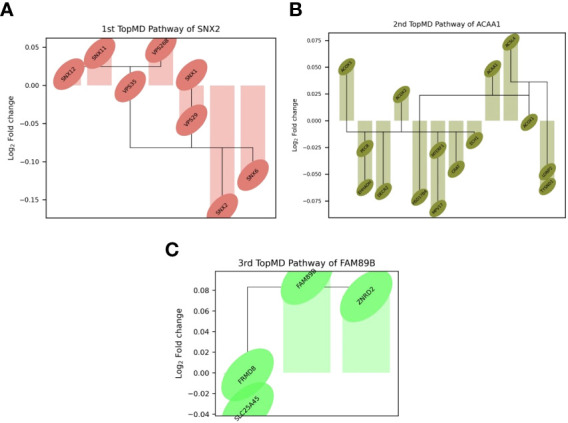
Differential expression of top genes in the top 3 pathways between patients admitted to ICU and not admitted to ICU of the training set. Connections represent known gene interactions according to STRING-db. **(A)** SNX2 - controlling epidermal growth factor receptor (EGFR) presentation, **(B)** ACAA1-peak pathway, representing peroxisome proliferator-activated receptor alpha (PPAR-α) signalling, **(C)** FAM89B-peak pathway, mediating transforming growth factor beta (TGF-β) signalling. Pathways and genes identified by topological data analysis, TopMD.

TopMD analysis identified pathways associated with ICU admission by defining and ranking pathways by their topological volume, the sum of normalised differential expression. The gene with the largest fold change was termed the peak-gene of the identified pathway. The top pathway had peak gene SNX2, associated with epidermal growth factor receptor (EGFR) signalling, followed by ACAA1, associated with Peroxisome proliferator-activated receptor alpha (PPAR-α) signalling and finally, FAM89B associated with Transforming growth factor beta (TGF-β) signalling (**Figure 3**). Additional peak genes and pathways are presented in **Supplementary Figure 1**. These consist of peak genes PHETA1, KEAP1, BAIAP2, TRAPPC6A, AGXT, HES1 and CDK5R1. Highlighting pathways such as phosphatidylinositol signalling, and glyoxylate and dicarboxylate metabolism (**Supplementary Figure 1**).

## Discussion

The emergence of the novel infectious agent SARS-CoV-2 has had a huge impact on healthcare systems worldwide and highlighted the importance of pandemic preparedness and management of limited healthcare resources. Here we demonstrate using retrospective analysis of gene expression data from patients hospitalised with COVID-19, at the point of admission, that there are markers that can predict the patient’s clinical outcome.

Like many other studies have previously identified, there were significant differences between clinical observations and physiological metrics for those who were and were not admitted to the ICU. In this study population, this included white blood cell count, neutrophil count, albumin, LDH, ferritin, CRP, IL-6, IL-33, oxygen saturation, NEWS, and consolidation of infiltrates (**Table 1**). This is in line with previous studies (17, 32). To further understand the host response in this study population and to determine whether mRNA signatures were able to predict ICU admission, a combination of topological analysis and machine learning was employed to identify genes and related pathways that predict disease.

To test the predictive nature of the model, data was split randomly into training and test datasets. There were differences in variables between the training and test cohorts (**Table 2**). Differences in measured variables are expected with high dimensional profiling of randomly split cohorts. The results of this study represent biological mechanisms which are consistent across the training and test cohorts, however, they are likely to be not the only mechanisms at play in driving COVID-19 disease severity, including those related to variables not balanced between the training and test cohorts.

COVID-19 gene expression prognosis studies are limited (33, 34). Scoring algorithm of molecular subphenotypes (SAMS) have been used to identify 50-gene risk profiles for COVID-19 which discriminate between mild and severe disease (33). Such profiles were able to predict ICU admission, the need for mechanical ventilation and mortality with an AUC of 0.77, 0.75 and 0.74 respectively. Immunophenotyping in addition to transcriptomic analysis on data derived from COVID-19 patients has led to the discovery of molecules that were associated with more severe disease, however, no AUC values were presented (34). In our analysis we ranked the top 30 most important genes with random forest, achieving an accuracy of 0.73 and ROC of 0.68, where FAM219A was identified as the most important variable for predicting ICU admission. FAM219A has been identified as a potential interactor with the SARS-CoV-2 M protein (35), however, the transcripts function is unknown.

TopMD analysis is an emerging topological data analysis (TDA) technology. When using high dimensional and noisy biological data sets, such as gene expression data, TDA approaches are particularly advantageous and have been successful in disease sub-phenotyping studies (25, 36–40). These approaches facilitate measurement of genes relative to their networks in disease context as opposed to the conventional differential abundance analysis, traditionally utilised in biomarker discovery. The TopMD algorithm was applied to gene expression data from COVID-19 patients at point of admission, with varying care trajectories. Our analysis shows that gene expression signatures in blood predict ICU admission. Gene expression signatures predictive of ICU admission were defined by machine learning and TopMD with accuracy: 0.73 and ROC: 0.68 and accuracy: 0.72 and ROC: 0.76 respectively. Topological analysis with TopMD improved the predictive model in comparison to the machine learning approach, demonstrating the advantages of considering the shape of data relative to underlying biological mechanisms above standard bioinformatic approaches which rely on statistical analysis of abundances of isolated molecules in vastly reduced, noisy, ‘omics datasets.

The TDA analysis of gene expression relative to pathways by TopMD acts as a global pathway analysis tool, defining patterns of differentially expressed genes with evidenced interactions. The top pathways differentially modulated between patients admitted to ICU and not admitted to ICU were 1^st^, SNX2-peak pathway, controlling epidermal growth factor receptor (EGFR) presentation, 2^nd^, ACAA1-peak pathway, representing peroxisome proliferator-activated receptor alpha (PPAR-α) signalling and 3^rd^, FAM89B-peak pathway, mediating transforming growth factor beta (TGF-β) signalling. (**Figure 2**). SNX2 was the top peak gene identified through TopMD analysis and is associated with EGFR signalling pathways. Dysfunctional EGFR signalling has been identified as a contributing factor to pulmonary fibrotic-like illness during SARS-CoV infections in animal models following the SARS-CoV pandemic in 2002, where authors speculated that inhibiting EGFR pathways would prevent fibrotic disease (41, 42). This is further supported by similar findings in SARS-CoV-2 infected patients, whereby EGFR was again found to be a regulator of pulmonary fibrosis (43). Inhibiting this pathway with nimotuzumab, a monoclonal antibody against EGFR, was found to decrease inflammatory markers and fibrosis associated with COVID-19 (44, 45). ACAA1; the peak gene of the second top pathway; is representative of PPAR-α signalling. PPAR-α signalling is a key mediator of inflammation, and like EGFR a potential marker for acute lung injury. Modulation of PPAR-α signalling by SARS-CoV-2 may alter lipid metabolism in the lung epithelial cells, contributing to lipotoxicity, inflammation and untoward respiratory effects (46). Therapeutics such as fenofibrate that target PPAR-α have been recommended to enter clinical trials (47). Where others have proposed that oleoylethanolamide (OEA), a high-affinity agonist to PPAR-α and ultramicronised palmitoylethanolamide (PEA), may have therapeutic effects by suppressing inflammatory responses (48, 49). Where PEA is also able to inhibit SARS-CoV-2 entry and replication (50). Interestingly, others have identified PPAR-α as a potential mediator neuroinflammation in COVID-19 (51). The third Top pathway had peak gene FAM89B, representing TGFβ signalling pathway, which is also associated with pulmonary fibrosis (52). TGFβ is a known regulator of immune reactions and its signalling is associated with fibrosis (53, 54). In the context of COVID-19, TGFβ gene signatures are observed in plasmablasts following seroconversion and is associated with a chronic immune reaction and severe disease (52). Within the ten pathways, peak gene KEAP1 was identified as a biomarker for ICU admission. KEAP1 is most well-known for its interaction with Nrf2 facilitating its ubiquitination, where exploiting this interaction to manage cytokine storms has been discussed in the context of COVID-19 (55, 56).

A key limitation of this study is that only one time point was considered in this analysis, although this was at the point of admission to hospital, which demonstrates its potential value as a POC tool, it does not consider the dynamic element of disease-course, future studies would benefit from gene expression measured at multiple time points. RNA sequencing can take a long time, however, with the third-generation sequencing platforms, rapid biomarker discovery and implementation at POC may be possible in the future. RNA sequencing at the bedside for personalised and precision medicine may not be an accessible solution for healthcare systems at this point in time, however, our data and analysis shows the potential use of sequencing data for prognosis. As sequencing costs continue to fall and accessibility to sequencing increases, this concept could progress to the bedside. In the case of retrospective analysis, useful pathways can also be identified informing future research and thus our understanding of disease.

Prognostic gene expression signatures identified here, upon further validation in independent cohorts, could be used to inform management of healthcare resources and improve outcomes of patients with COVID-19. Gene expression signatures measured in global RNAseq transcriptomics data could be applied across health and disease for precision medicine.

## Data availability statement

The datasets presented in this study can be found in online repositories. The names of the repository/repositories and accession number(s) can be found below: European Genome-Phenome Archive, EGAS00001005971.

## Ethics statement

The studies involving human participants were reviewed and approved by South Central Hampshire A Research Ethics Committee. The patients/participants provided their written informed consent to participate in this study.

## Author contributions

TC and DB conceptualised the study. SP and NB screened and recruited the patients and collected the data in the CoV-19POC trials. RP-R and CH sample processing and experiments. RP-R, XD, AG, JS, and JH performed data analysis. RP-R, AG, JS, and JH drafted the article, and editing. All authors read and approved the final manuscript.

## Funding

The author(s) disclosed receipt of the following financial support for the research, authorship, and/or publication of this article: the CoV-19POC trial was funded by University Hospital Southampton Foundation Trust (UHSFT). In addition, the CoV-19POC trials were supported by the NIHR Southampton Clinical Research Facility and NIHR Southampton Biomedical Research Centre (BRC). JLe was supported by a PhD studentship from the NIHR Southampton BRC (no. NIHR-INF-0932). NB was supported by the NIHR Clinical Lecturer scheme. JH, RP-R, CH, and XD were supported by the US Food and Drug Administration Medical Countermeasures Initiative (no 75F40120C00085) awarded to JH. MP was supported by a Sir Henry Dale Fellowship from Welcome Trust and The Royal Society (no. 109377/Z/15/Z). TC was supported by a NIHR Post-Doctoral Fellowship (no. 2016-09-061). DB and her laboratory are supported by a NIHR Research Professorship (no. RP-2016-07-011). TopMD, the University of Southampton and the University of Liverpool are members of the DRAGON consortium, which received funding from the Innovative Medicines Initiative 2 Joint Undertaking (JU) under grant agreement no 101005122. The funders had no role in study design, data collection and analysis, decision to publish, or preparation of the manuscript.

## Acknowledgments

The authors would like to acknowledge and gives thanks to the patients who kindly participated in this study and to all the clinical staff at University Hospital Southampton Foundation Trust who cared for them. The DRAGON consortium is a group of high-tech SMEs, academic research institutes, biotech and pharma partners, affiliated patient-centered organisations and professional societies aiming to apply artificial intelligence for improved and more rapid diagnosis and prognosis in COVID-19.) Further details may be found at https://www.imi.europa.eu/projects-results/project-factsheets/dragon.

## Conflict of interest

TC has received speaker fees, honoraria, travel reimbursement, and equipment and consumables free of charge for the purposes of research from BioFire diagnostics LLC and BioMerieux. TC has received discounted equipment and consumables for the purposes of research from QIAGEN. TC has received consultancy fees from Biofire diagnostics LLC, BioMerieux, Synairgen research Ltd, Randox laboratories Ltd and Cidara therapeutics. TC has been a member of advisory boards for Roche and Janssen and has received reimbursement for these. TC is member of two independent data monitoring committees for trials sponsored by Roche. TC has previously acted as the UK chief investigator for trials sponsored by Janssen. TC is currently a member of the NHSE COVID-19 Testing Technologies Oversight Group and the NHSE COVID-19 Technologies Validation Group. JS is a founding director, CEO, employee, and shareholder in TopMD Precision Medicine Ltd. FS is a founding director, CTO, employee, and shareholder in TopMD Precision Medicine Ltd. PS is a founding director, employee and shareholder in TopMD Precision Medicine Ltd. AG is an employee and shareholder in TopMD Precision Medicine Ltd. RP-R is an employee at TopMD Precision Medicine Ltd.

The remaining authors declare that the research was conducted in the absence of any commercial or financial relationships that could be construed as a potential conflict of interest.

## Publisher’s note

All claims expressed in this article are solely those of the authors and do not necessarily represent those of their affiliated organizations, or those of the publisher, the editors and the reviewers. Any product that may be evaluated in this article, or claim that may be made by its manufacturer, is not guaranteed or endorsed by the publisher.
