# Supplementary material for: Blood gene expression predicts intensive care unit admission in hospitalised patients with COVID-19
Source: Front Immunol. 2022 Sep 20;13:988685. doi: 10.3389/fimmu.2022.988685 (PMC9530807; doi:10.3389/fimmu.2022.988685)
Supplement: Supplementary file 1 [file DataSheet_1.docx]

##
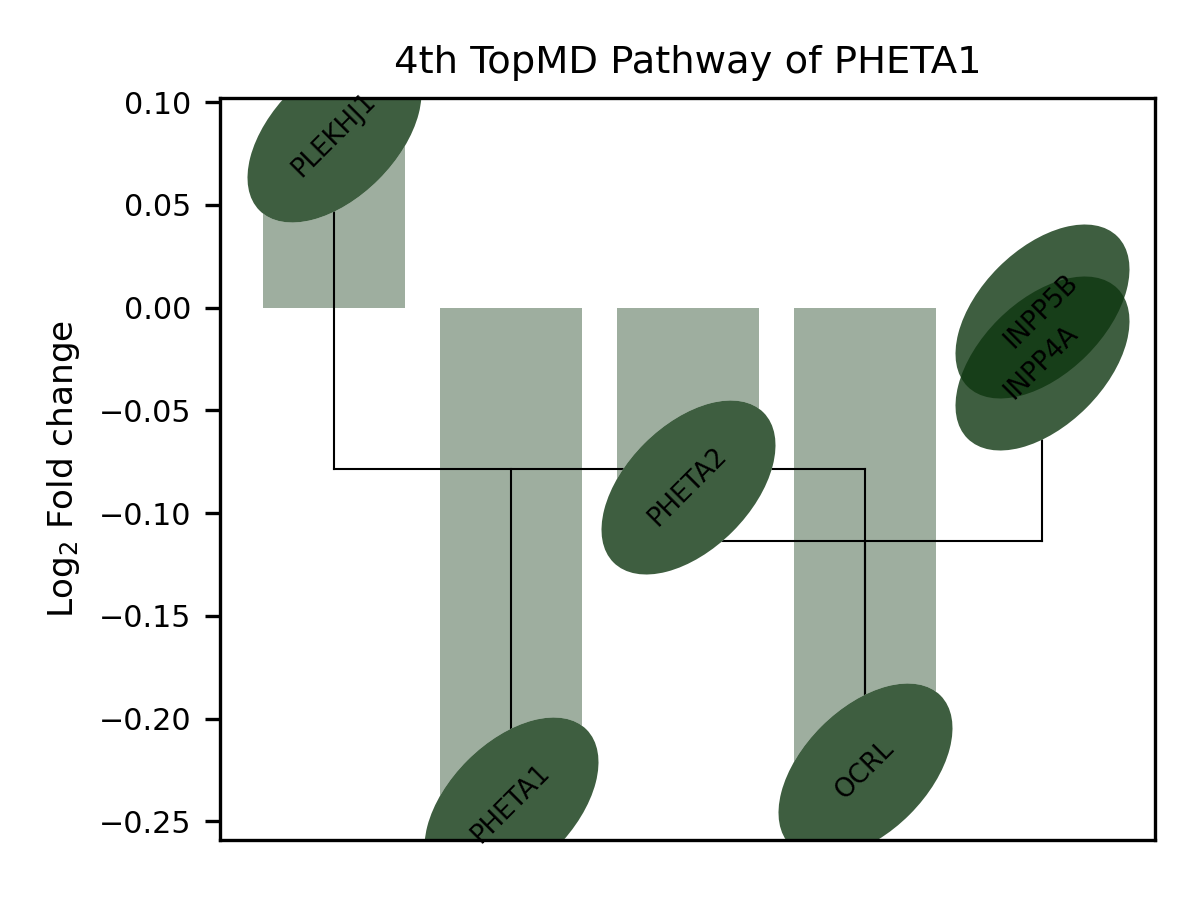

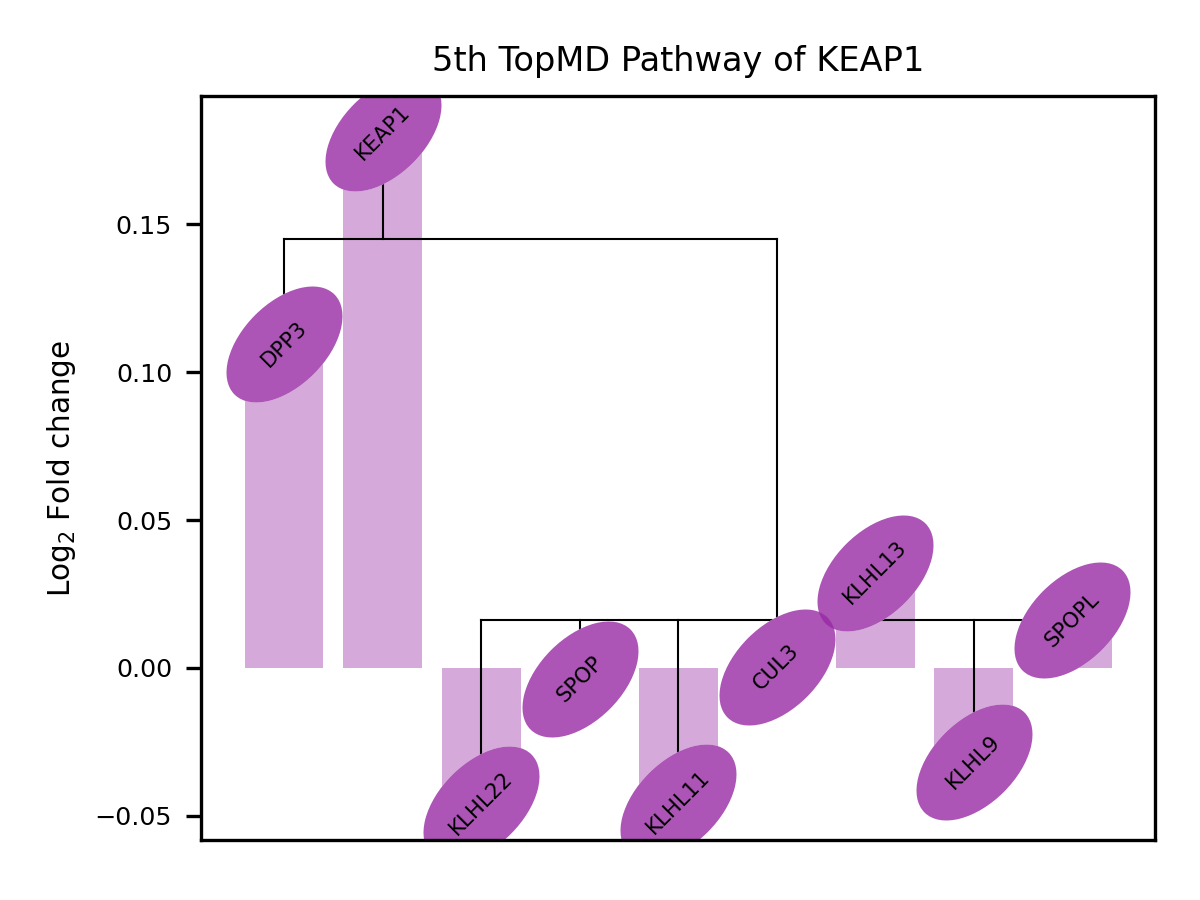
Supplementary figures

##
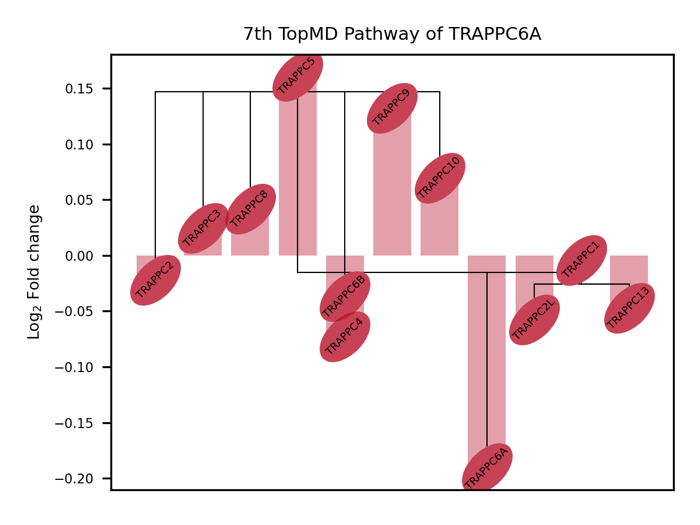


##
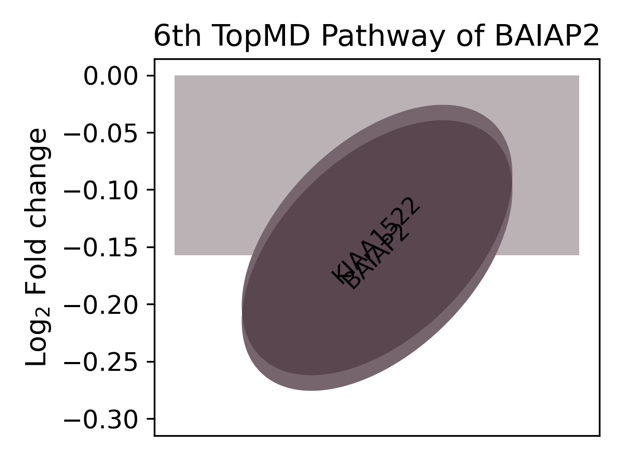


##
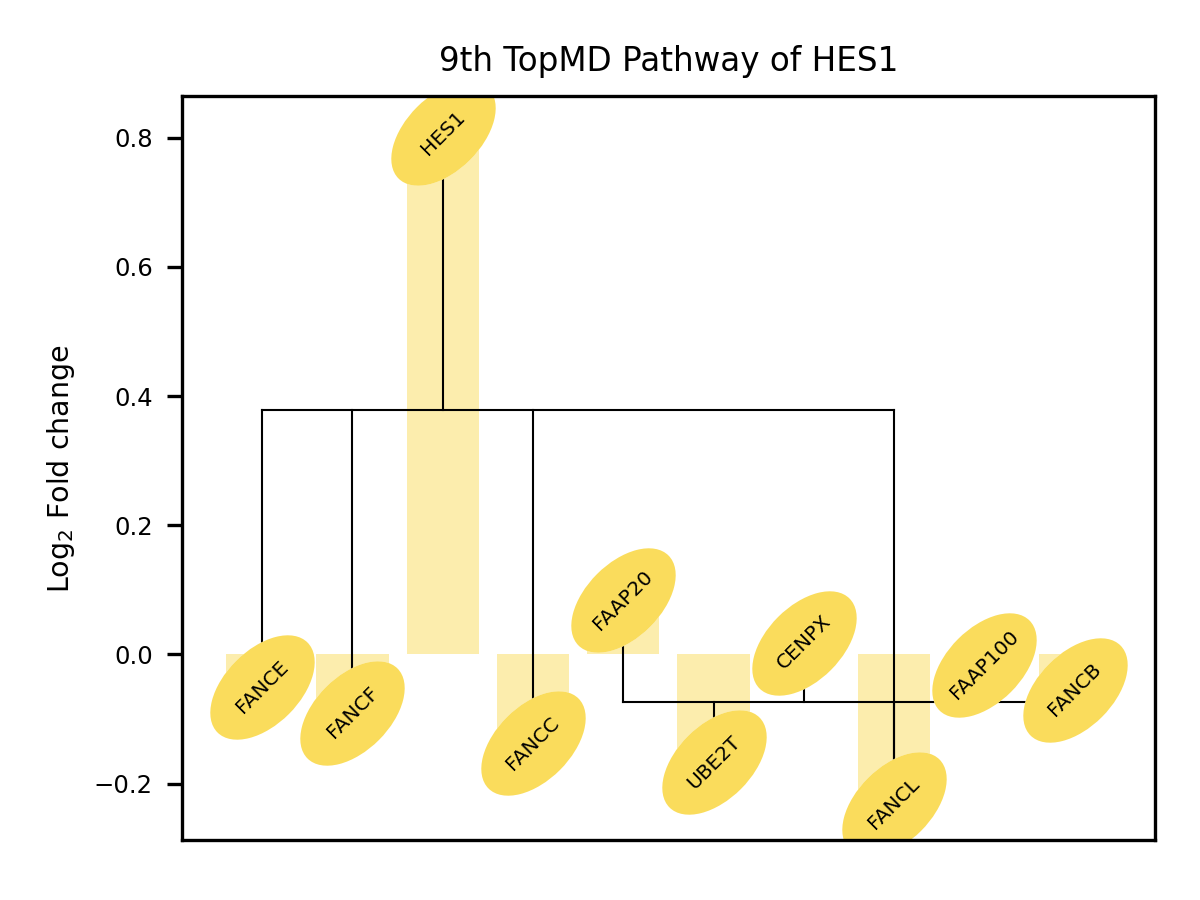


##
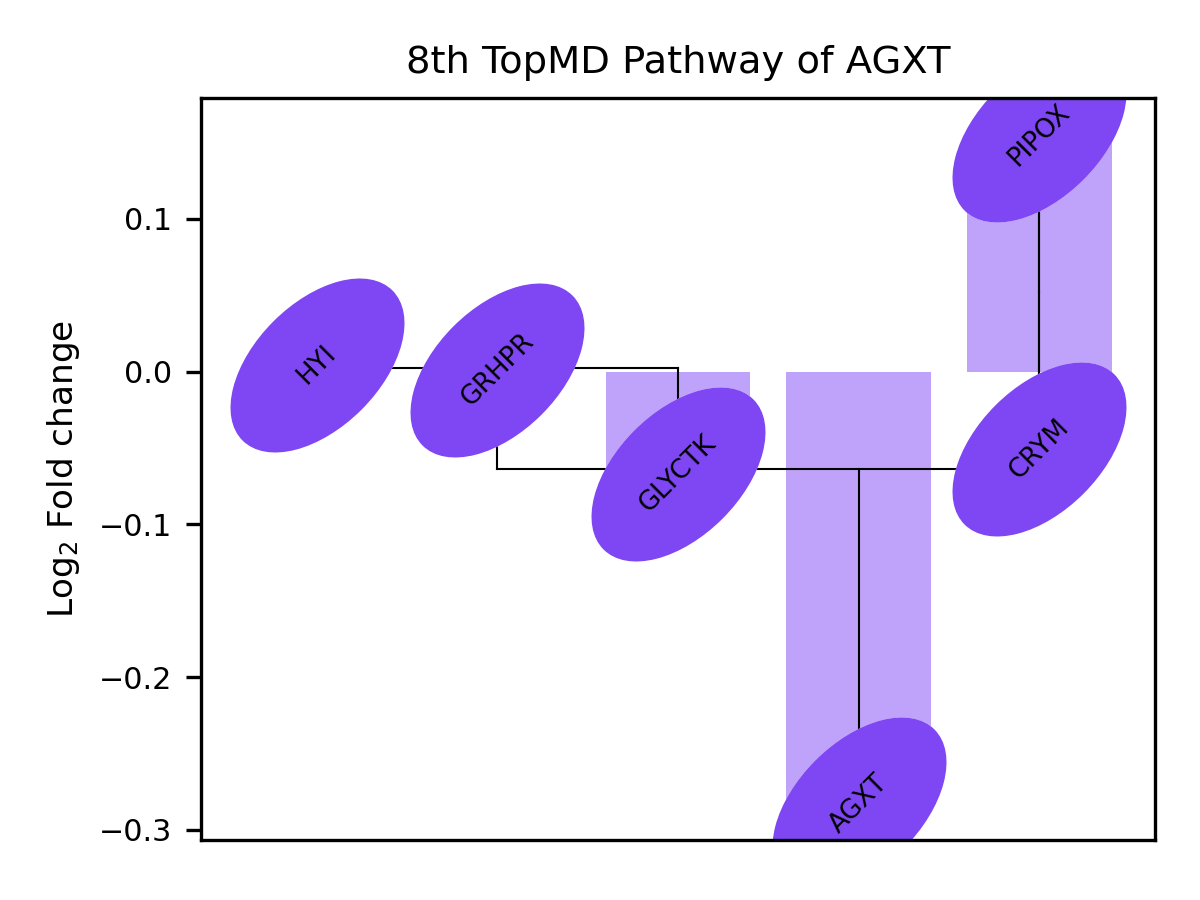


##
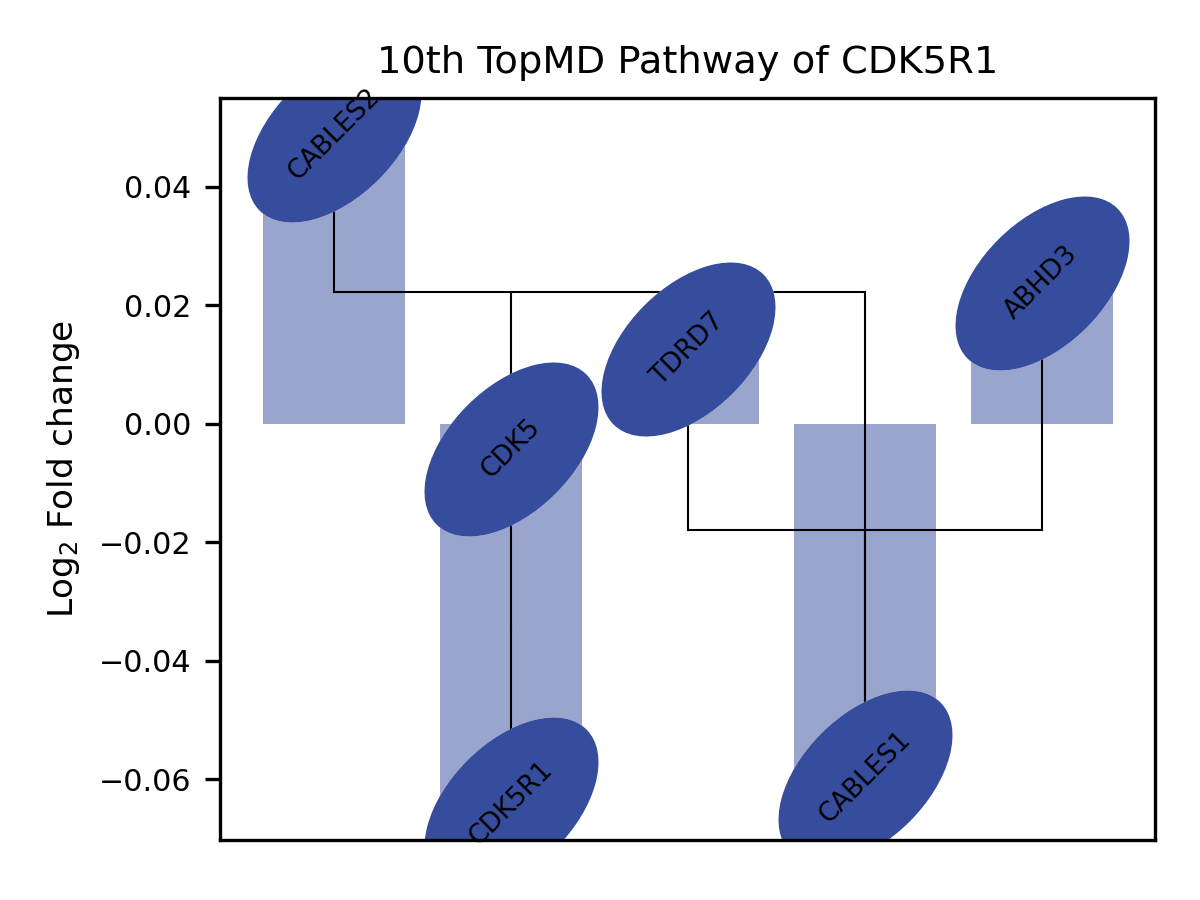


**Supplementary figure 1:** Differential expression of top genes in the remaining 7 pathways out of the top 10, between patients admitted to ICU and not admitted to ICU of the training set. Pathways and genes identified by topological data analysis, TopMD.
